# CircITGA7 Suppresses Gastric Cancer Progression Through miR-1471/MTDH Axis

**DOI:** 10.3389/fcell.2021.688970

**Published:** 2021-08-24

**Authors:** Haifeng Jin, Zheng Wu, Bibo Tan, Zhen Liu, Binqian Zhang

**Affiliations:** ^1^Department of Gastroenterology, The 980th Hospital of the PLA Joint Logistics Support Force (Primary Bethune International Peace Hospital of PLA), Shijiazhuang, China; ^2^Department of Immuno-Oncology, Fourth Hospital of Hebei Medical University, Shijiazhuang, China; ^3^Department of General Surgery, Fourth Hospital of Hebei Medical University, Shijiazhuang, China; ^4^Department of Clinical Medicine, Chongqing Engineering Research Center of Pharmaceutical Sciences, Chongqing Medical and Pharmaceutical College, Chongqing, China

**Keywords:** CircITGA7, gastric cancer, miR-1471, MTDH, The Cancer Genome Atlas

## Abstract

In recent years, there have been reports about the involvement of circular RNAs (circRNAs) in the pathogenesis of gastric cancer (GC), but the molecular mechanism in cell proliferation, invasion, and migration is still unclear. Based on The Cancer Genome Atlas (TCGA) database, we analyzed differentially expressed circRNAs between GC and non-tumor tissues. Gene Ontology and Kyoto Encyclopedia of Genes and Genomes enrichment analysis were used to clarify the functional role in GC. Here, we showed that circITGA7 was lowly expressed in GC tissues based on the TCGA database. *In vitro*, silencing the expression of circITGA7 increased cell proliferation and metastasis, whereas overexpression did the opposite. Mechanistically, miR-1471 has circITGA7 as a sponge, and miR-1471 has metadherin (MTDH) as a target gene. Consequently, functional analysis showed that the tumor suppressor effect of circITGA7 was the result of regulating the miR-1471/MTDH axis. Overall, the circITGA7/miR-1471/MTDH signaling pathway may play a crucial role in GC, providing a new potential mechanism involved in GC progression.

## Introduction

Although both morbidity and mortality rates have declined significantly over the past few decades, gastric cancer (GC) remains to be a very common cancer and the primary inducer of death globally (Bertuccio et al., 2009). The results of the present study suggest that GC has a high mortality rate, especially after diagnosis (Gu et al., 2020b). The survival rate of GC patients is low, and the prognosis is also poor (Akoh and Macintyre, 1992; Allgayer et al., 1997). At present, surgery is the only radical treatment (Van Cutsem et al., 2016). With the advancement of surgical technology and the implementation of traditional radiotherapy, chemotherapy, and neoadjuvant therapy, the 5-year survival rate of early diagnosed GC has been enhanced more than 95% (Crew and Neugut, 2006). However, the low rate of early diagnosis means that most patients are so advanced by the time they are diagnosed that the best time for surgery is missed (Pasechnikov et al., 2014). Therefore, the most effective methods for the treatment of advanced GC are neoadjuvant chemoradiotherapy and molecular targeted therapy (Yang et al., 2014; Aoyama and Yoshikawa, 2017). Although researches have shown that in the timing and treatment of GC, a variety of methods are effective, but no one standard treatment is widely accepted, it also involves the best timing of chemotherapy, the benefits of radiotherapy, the efficacy of chemotherapy, lymph node dissection to minimize the scope of the best treatment, and so on (Alberts et al., 2003). In view of the poor prognosis of patients, it is essential to find early diagnosis and convenient and timely access to effective treatment.

Circular RNA (circRNA) is a long non-coding RNA molecule with a polar, covalently close continuous ring without a poly-A tail. Many circRNAs are highly conserved and have specific expression patterns, usually not related to host gene expression (Patop et al., 2019). CircRNA is a new type of non-coding tumor genome (Patop and Kadener, 2018). More and more evidences indicate the possible function of circRNAs in developing a number of diseases, including the risk of atherosclerotic vascular diseases and neurological diseases, viral diseases, osteoarthritis, and diabetes (Xu et al., 2018). However, the correlation of most functions has not yet been found. New evidence shows that there are thousands of circRNAs in mammalian cells that bind to microRNAs (miRNAs) or other molecules to mediate gene expression at the transcriptional or posttranscriptional level, thereby inhibiting its function (Rong et al., 2017). CircRNA-UBAP2 can be used as a ceRNA of spongy miR-382-5p in ovarian cancer, increasing the expression level of PRPF8, promoting the proliferation of ovarian cancer cells, and inhibiting cell apoptosis (Xu et al., 2020). GC has also been reported. Circ_0081143 altered migration, invasion, and epithelial/mesenchymal transformation of GC through the miR-497-5p/EGFR axis (Tang et al., 2020). Through modulating the miR-646/LRP6 axis, Circ_0000527 promotes proliferation and metastasis of retinoblastoma cells (Zhang et al., 2020b). CircRNA, being a diagnostic and prognostic biomarker, may be a therapeutic target for individualized medicine in the future (Meng et al., 2017).

Competitive endogenous RNA (ceRNA) is a transcript that can be mutually regulated at the posttranscriptional level by competing shared miRNAs (Gu et al., 2020d). The ceRNA network connects the function of protein-coding mRNA with the function of non-coding RNA (such as miRNA, long non-coding RNA, pseudogene RNA, and circRNA). circRNA has been shown to interact with miRNA as a ceRNA to regulate target gene expression and participate in tumorigenesis. The construction of circRNA-related ceRNA networks in breast cancer reveals that circRNA is related to the progression and prognosis of breast cancer. The identification of ceRNA network analysis in bladder cancer is used to predict the prognosis of bladder cancer as circRNA biomarkers.

In this study, we first evaluated how ITGA7 expressed in GC using TGGA database. Then reverse transcription–quantitative polymerase chain reaction (RT-qPCR) assay revealed that circITGA7 was downregulated in GC tissues and cells. Further functional researches proved that circITGA7 could suppress the development of GC. In addition, circITGA7 may participate in the occurrence and progression of GC through regulating miR-1471 and target gene metadherin (MTDH).

## Materials and Methods

### Data Source

RNA sequencing data of GC, also known as stomach adenocarcinoma (STAD), including 211 normal samples and 408 tumor samples, were obtained from The Cancer Genome Atlas (TCGA,^1^) database (Gu et al., 2021a).

### Differentially Expressed CircRNAs Analysis

The analysis of differentially expressed circRNAs in TCGA STAD tumor samples in comparison with normal samples was accomplished by using the limma package. Differentially expressed circRNAs’ *P* values were counted by *t* test during analysis. A cutoff criterion was set as | log2FC| ≥ 1 and *P* < 0.05 (Shi et al., 2018; Zhang et al., 2020a).

### Gene Ontology and Pathway Enrichment Analyses

Gene Ontology (GO) is used for gene annotation, including three categories: cell component (CC), biological process (BP), and molecular function (MF) (Gu et al., 2020c). The Kyoto Encyclopedia of Genes and Genomes (KEGG) is a database for associating gene sets with related pathways (Gu et al., 2021b). DAVID (Database for Annotation, Visualization and Integrated Discovery) is an online device to operate GO annotation and KEGG pathway enrichment analyses.

### Module Analysis

Package Molecular Complex Detection (MCODE) of Cytoscape software was employed to aid in the analysis of the most dominant clustering module. Then, DAVID was adopted to analyze the GO term that was enriched by DEGs in different modules.

### Patient Tissue Samples and Cell Line

The samples of GC patients, as well as the corresponding normal tissues, were collected from our hospital. Each participant has provided the written informed consent, and our Hospital Ethics Committee has approved all the procedures. GES-1, BGC-823, NCI-N87, SGC7901, and AGS cells were used. In Dulbecco modified eagle medium (Gibco, NY, United States) supplemented with 10% fetal bovine serum (Gibco, United States) at 37°C in a 5% CO_2_ incubator, all cell types were cultured.

### RT-qPCR

Trizol reagent (Invitrogen, United States) was employed to obtain total RNA of GC cell and tissues. Then, the cDNA was obtained from total RNA using the Bestar^TM^ qPCR RT kit. Based on the guidance of the manufacturer, RT-PCR reactions were applied using the ABI 7500 system with Bestar^TM^ qPCR MasterMix.

### RNA Interference

The siRNA against circITGA7, miR-1417 mimics, anti-miR1417, and controls were produced by Genepharm. The sequence of circITGA7 was cloned into the pcDNA3.1 vector to obtain overexpressed circITGA7. In six-well plates, SGC7901 and AGS cells were cultured, and then they were transfected with siRNA using LipoFiter transfection reagent (Hanbio) following the guidance of the manufacturer.

### Dual-Luciferase Activity Assay

The insertion of the circITGA7 and MTDH-3′ UTR fragment that contained the wild-type (WT) or mutant (MUT) miR-1471–binding site into the pGL3 vector (Promega, United States) was completed to construct the circITGA7 WT or MUT reporter vector.

For the dual-luciferase reporter assay, SGC7901 and AGS cells were seeded and then cotransfected with miRNAs and vector reporter plasmids with Lipofectamine 2000 (Invitrogen). Then, according to previous reports, dual-luciferase reporter assay was employed to evaluate luciferase activity. Renal luciferase activity was normalized to firefly luciferase activity.

### Migration Assay

Cell migration assays were conducted with 8-μm pore size Transwell chamber (Corning). Cancer cells (2 × 10^4^) in complete medium were added to the lower chamber, and those in 100 μL serum-free medium were added to the upper chamber. After being incubated for 24 h, the migrated cells were counted under a microscope according to a previous report.

### Invasion Assay

A 24-well Transwell chamber was used to conduct the cell invasion assay. Transfected cells (5 × 10^4^ cells/well) were suspended in serum-free medium and then added to superluminal or matrix gel-coated Transwell inserts. The medium, which contained 10% fetal bovine serum, was added to the lower chamber. After 48 h of incubation, the migrated cells were counted under a microscope according to a previous report.

### CCK-8 Assay

At a density of 2 × 10^3^ cells/well, GC cells were seeded. CCK-8 reagent (Dojindo Chemical Laboratory, Kumamoto, Japan) was added 3 h before the end of the experiment. The absorbance was measure with a microplate reader at 450 nm (Gu et al., 2020a).

### Statistical Analysis

In this study, the data are presented as mean ± SEM (Pan et al., 2017). GraphPad Prism version 7.0 software was employed to operate two-way analysis of variance or Student *t* test (Chen et al., 2021). *P* value, which was less than 0.05, was taken as having statistical significance.

## Results

### ITGA7 Was Lowly Expressed in GC Tissues

**FIGURE 1:**
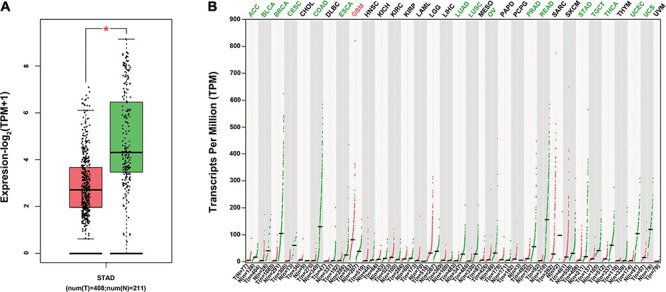
The expression of ITGA7 in various cancer tissues using TCGA. **(A)** ITGA7 was lowly expressed in STAD *P < 0.05. **(B)** ITGA7 lowly expressed in many cancer tissues but highly expressed in GBM.

To investigate the specific role of ITGA7 in the development of GC, we first evaluated how ITGA7 expressed in GC using TGGA database, and it was revealed that ITGA7 was expressed lowly in STAD (Figure 1A). Besides, in many other tumors, such as rectum adenocarcinoma, colon adenocarcinoma, and breast invasive carcinoma (BRCA), ITGA7 was also lowly expressed (Figure 1B). However, ITGA7 was highly expressed in glioblastoma multiforme (GBM) tissues in comparison with adjacent normal tissues.

### GO Enrichment and Pathway Analysis

**FIGURE 2:**
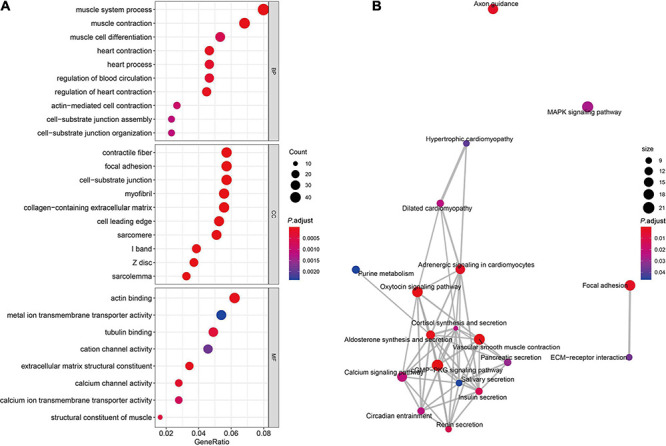
GO enrichment and pathway analysis. **(A)** Significantly enriched pathways of the DEGs in BP, MF, and CC were revealed by GO analysis. DEGs, differentially expressed genes; BP, biological process; MF, molecular function; CC, cellular component; GO, Gene Ontology. **(B)** The complex interaction network among the significantly enriched KEGG pathways.

In order to further understand the functional association of differentially expressed circRNAs in STAD, we used DAVID software to perform GO analysis and KEGG analysis. As Figure 2A shows, the results demonstrated that the significantly enriched GO terms in BP included muscle contraction, muscle system process, muscle cell differentiation, heart process, heart contraction, regulation of heart contraction, regulation of blood circulation, actin-mediated cell contraction, cell–substrate junction assembly, and cell–substrate junction organization. The significantly enriched GO terms in CC contained contractile fiber, focal adhesion, cell–substrate junction, myofibril, collagen-containing extracellular matrix, cell leading edge, sarcomere, I band, Z disk, and sarcolemma. The significantly enriched GO terms in MF comprised actin binding, tubulin binding, cation channel activity, extracellular matrix structural constituent, metal ion transmembrane transporter activity, calcium-channel activity, extracellular matrix structural constituent, calcium ion transmembrane transporter activity, and structural constituent of muscle. Furthermore, KEGG analysis demonstrated the association of these differentially expressed circRNAs with cGMP-PKG signaling pathway, the vascular smooth muscle contraction, axon guidance, aldosterone synthesis and secretion, oxytocin signaling pathway, adrenergic signaling in cardiomyocytes, focal adhesion, renin secretion, insulin secretion, calcium signaling pathway, dilated cardiomyopathy, cortisol synthesis and secretion, circadian entrainment, mitogen-activated protein kinase signaling pathway, pancreatic secretion, extracellular matrix–receptor interaction, hypertrophic cardiomyopathy, salivary secretion, purine metabolism (Figure 2B).

### Module Analysis

**FIGURE 3:**
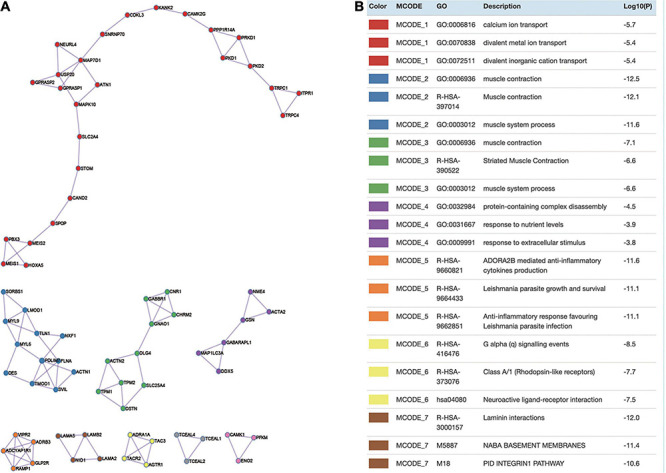
Module analysis. **(A)** Identify hub genes from the protein–protein interaction (PPI) network using MCODE algorithm. Each color denotes a cluster detected by MCODE. **(B)** GO enriched by DEGs in different modules.

Being one of the features of the protein–protein interaction (PPI), the network module could also provide specific and significant biological information. As shown in Figure 3, there were in total nine modules detected and constructed by MCODE. Then, we further performed GO term enrichment analysis of some module. The results showed that module 1 was involved in divalent inorganic cation transport, divalent metal ion transport, and calcium ion transport; module 2 was involved in muscle system process and muscle contraction; module 3 was involved in muscle system process, striated muscle contraction, and muscle contraction; module 4 was involved in protein-containing complex disassembly, response to extracellular stimulus, and response to nutrient levels; module 5 was involved in anti-inflammatory response favoring *Leishmania* parasite infection, *Leishmania* parasite growth and survival, and ADORA2B-mediated anti-inflammatory cytokine production; module 6 was involved in class A/1 (rhodopsin-like receptors), G alpha (q) signaling events, and neuroactive ligand–receptor interaction; module 7 was involved in laminin interactions, NABA basement membranes, and PID integrin1 pathway.

### The Expression of CircITGA7 Was Remarkably Decreased in GC

**FIGURE 4:**
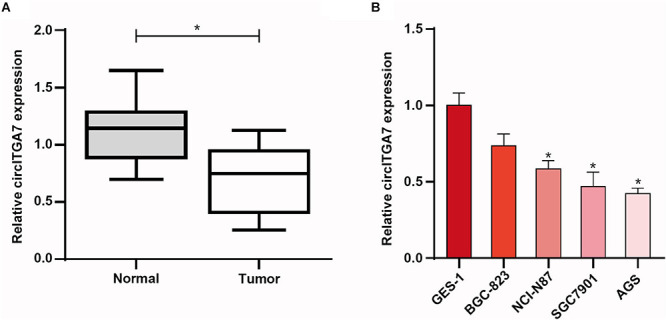
The expression of circITGA7 was remarkably decreased in gastric cancer. **(A)** Compared to normal tissues, CircITGA7 expressed significantly lower in GC tissues *P < 0.05. **(B)** Expressions of circITGA7 in gastric tumor cell lines and gastric epithelial cell line *P < 0.05.

To explore the role of circRNAs in GC, we first compared the expressions of circITGA7 between normal tissues and GC tissues. The data showed that compared to normal tissues, circITGA7 expressed significantly lower in GC tissues (Figure 4A). Furthermore, we examined the expressions of circITGA7 in GC cell lines (BGC-823, AGS SGC7901, and NCI-N87), and one healthy gastric epithelial cell (GES-1) was set as negative control. qRT-PCR assays confirmed that in healthy gastric cells, the expression levels of circITGA7 were lower in GC cells lines, especially in SGC7901 and AGS cells (Figure 4B).

### CircITGA7 Suppressed the Development of GC *in vitro*

**FIGURE 5:**
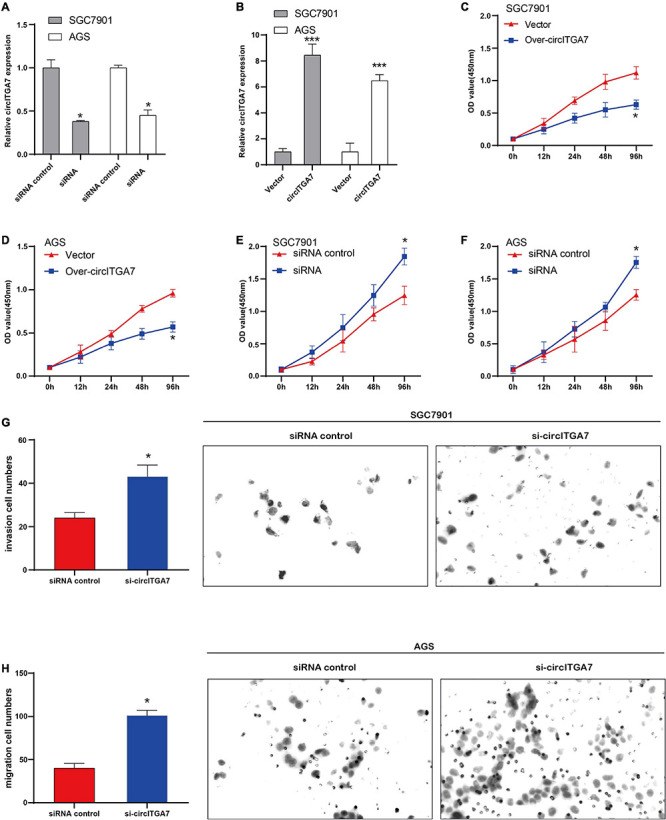
CircITGA7 suppressed the development of GC *in vitro*. **(A)** The expressions of circITGA7 were remarkably downregulated in SGC7901 and AGS cells transfected with siRNA *P < 0.05. **(B)** The expressions of circITGA7 were remarkably upregulated in SGC7901 and AGS cells transfected with circITGA7 ***P < 0.001. **(C,D)** The cell proliferation in circITGA7-overexpressed SGC7901 and AGS cells was notably decreased *P < 0.05. **(E,F)** The cell proliferation in circITGA7 lowly expressed SGC7901 and AGS cells was notably decreased *P < 0.05. **(G)** The invasion ability in circITGA7 lowly expressed SGC7901 cells was significantly decreased. **(H)** The migration ability in circITGA7 lowly expressed AGS cells was significantly decreased.

We regulated high circITGA7’s expression in SGC7901 and AGS cells to explore its role in GC. In order to detect the circITGA7 expression, qRT-PCR was applied. The results showed that compared to control cells, the expressions of circITGA7 were remarkably downregulated in cells transfected with siRNA (Figure 5A) and upregulated in cells transfected with circITGA7 overexpression plasmid (Figure 5B). Then, it was shown that cell proliferation in circITGA7-overexpressed SGC7901 and AGS cells was significantly decreased (Figures 5C,D). Similarly, in circITGA7 lowly expressed cells, cell proliferation was significantly increased (Figures 5E,F). In Transwell assays, we added si-ITGA7 in SGC7901 and AGS cells to conduct invasion and migration assays, respectively. The results demonstrated that in circITGA7-downregulated cells, the abilities to invade and migrate were enhanced (Figures 5G,H).

### CircITGA7 Was a Sponge for miR-1471

**FIGURE 6:**
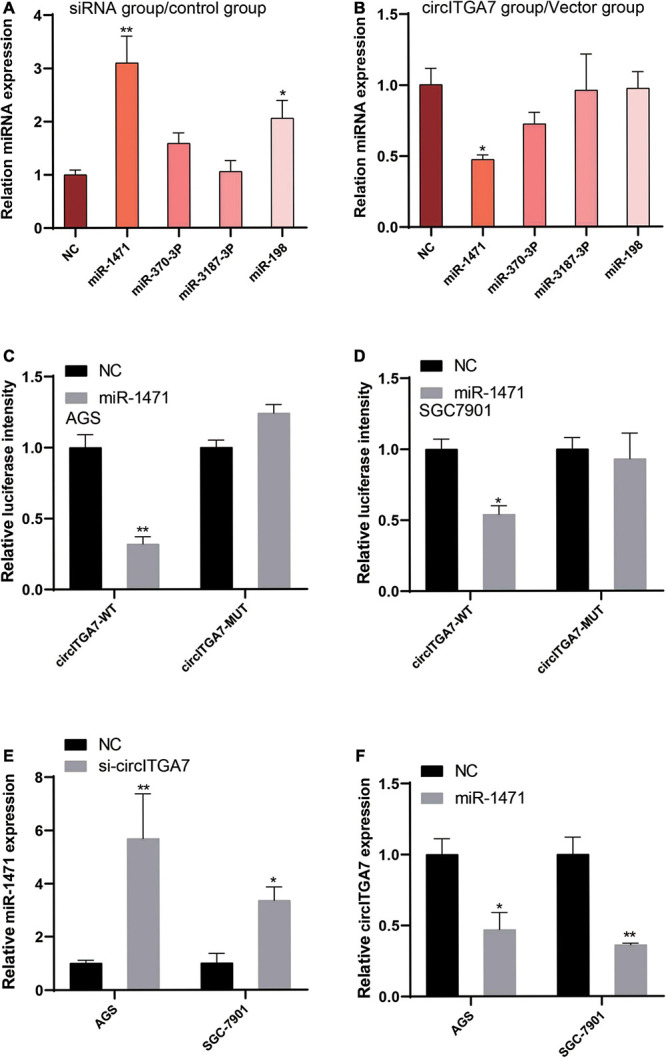
CircITGA7 was a sponge for miR-1471. Expressions level of four miRNAs in AGS cells transfected with **(A)** siRNA or **(B)** circITGA7 *P < 0.05, **P < 0.01. Overexpressed miR-1471 did not attenuate the luciferase activity driven by circITGA7 Mut, but significantly attenuated those driven by circITGA7 WT in **(C)** AGS and **(D)** SGC7901 cells *P < 0.05, **P < 0.01. **(E)** Increased miR-1471 expression in AGS and SGC7901 cells transfected with si-circITGA7 *P < 0.05, **P < 0.01. **(F)** Decreased circITGA7 expression in AGS and SGC7901 cells transfected with miR-1471 mimics *P < 0.05, **P < 0.01.

To further explore the potential mechanism of circITGA7 in GC, we predicted the target miRNAs of circITGA7. We used miRBase^2^ and circInteractome^3^ to analyze the potential targets of circITGA7. The ceRNA network analysis showed circITGA7 interacted with four miRNAs (miR-1471, miR-370-3p, miR-3187-3p, and miR-198). Thus, we examined the expressions of these four miRNAs in AGS cells transfected with siRNA or circITGA7-overexpressed plasmid, and the results are shown in Figures 6A,B. MiR-1471 expressed differently than the other three miRNAs. Next, to explore the interaction between miR-1471 and circITGA7 in SGC7901 and AGS cells, dual-luciferase reporter assay was conducted. Results revealed that miR-1471 mimics significantly attenuated the luciferase activity driven by circITGA7 WT in both AGS and SGC7901 cells, whereas miR-1471 mimics did not attenuate those driven by circITGA7 Mut (Figures 6C,D). Then, we observed the expression of miR-1471 in SGC7901 and AGS cells transfected with sicircITGA7. The data showed miR-1471 had an increased expression (Figure 6E). We also observed the expression of circTIGA7 in miR-1471–overexpressed SGC7901 and AGS. The results showed a decreased circITGA7 expression (Figure 6F). Therefore, there existed a negative relation between miR-1471 and circITGA7.

### MTDH Targeted by miR-1471 Reversed the Effect of CircITGA7

**FIGURE 7:**
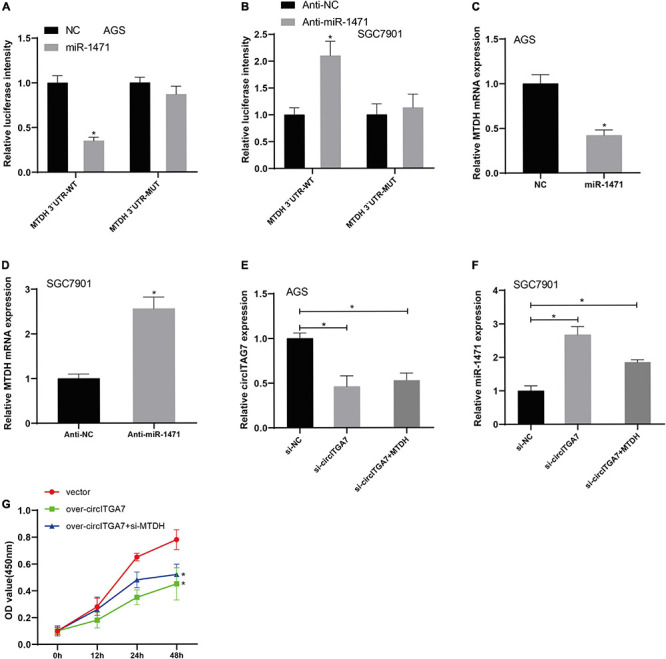
MTDH targeted by miR-1471 reversed the effect of circITGA7. **(A)** The luciferase activity driven by MTDH 3′ UTR-WT in AGS cells was remarkably reduced by overexpressed miR-1471 *P < 0.05. **(B)** The luciferase activity driven by MTDH 3′ UTR-WT in SGC7901 cells was remarkably enhanced by lowly expressed miR-1471 *P < 0.05. **(C)** Decreased MTDH expression in AGS cells transfected with miR-1471. *P < 0.05. **(D)** Increased MTDH expression in SGC7901 cells transfected with anti– miR-1471. *P < 0.05. **(E)** MTDH partially reversed the downregulation of circITGA7 in AGS cell transfected with si-circITGA7 *P < 0.05. **(F)** MTDH partially reversed the upregulation of miR-1417 in SGC7901 cell transfected with si-circITGA7 *P < 0.05. **(G)** MTDH partially reverse the decreased cell proliferation activity by circITGA7 overexpression *P < 0.05.

According to the results of TargetScan 7.2^4^, MTDH was identified as miR-1471’s target gene. We have established the plasmids of MTDH WT and MUT recombinant luciferase plasmid to verify the interaction between miR-1471 and MTDH. Using dual-luciferase reporter experiments, we revealed that miR-1471 mimics remarkably reduced the expression of MTDH 3′ UTR-WT–driven luciferase activity in AGS cells (Figure 7A), whereas anti–miR-1471 enhanced luciferase activity in SGC7901 cells (Figure 7B). Then, we detected the levels of MTDH in SGC7901 and AGS cells transfected with miR-1471 and anti–miR-1471, respectively. The assays indicated that there existed a negative relation between MTDH and miR-1471 in GC cells (Figures 7C,D). We explored the effects of MTDH on SGC7901 and AGS cells treated with circITGA7 siRNAs to better clarify the significant function of MTDH in GC cell lines. MTDH partially reversed the downregulation of circITGA7 in AGS cell (Figure 7E), as well as reversed upregulation of miR-1471 in SGC7901 cell (Figure 7F) induced by circITGA7 siRNAs. In functional assays, we found that circITGA7 overexpression’s inhibition of SGC7901 cell proliferation activity was reversed with MTDH (Figure 7G). Therefore, it could be verified that MTDH had a positive relation with circITGA7 in GC cells, and circITGA7 affected GC progression through miR-1471/MTDH axis.

## Discussion

As we all know, GC is a very common tumor in the digestive system, which may be related to dietary habits, geographical environment, genes, and so on (Kim et al., 2014). At present, with the progress of pathological and biochemical diagnosis technology and surgical resection technology, the success rate of cancer treatment and the survival rate of patients after surgery have been significantly improved (Crew and Neugut, 2006; Yuan et al., 2017; Gu et al., 2018; Gu and Chen, 2020; Bao et al., 2021). Nevertheless, the number of GC deaths is increasing year by year worldwide, especially in East Asia (Rahman et al., 2014). Therefore, to explore the treatment of GC is imminent. Previously, Li et al. reported that Has_CIRC_0000096 influenced GC cells’ growth and migration by regulating CDK6, cyclin D1, MMP-9, and MMP-2. The results showed that circARKT3 derived from exons 8, 9, 10, and 11 of AKT3 gene could promote GC cell DNA damage repair and inhibit cell apoptosis (Li et al., 2017). In addition, Zhang et al. (2019) proved that circNRIP1 affected the expression level of AKT1 through miR-149-5p and finally acted as a tumor promoter in GC. Based on ITGA7 microarray studies, we found that ITGA7 was significantly low-regulated in many cancers including GC. At the same time, the enrichment analysis showed the close association between differentially expressed circRNAs’ biological functions and muscle process, which is in line with previous study. Being a complex syndrome, sarcopenia is defined as progressive and systemic loss of skeletal muscle mass and strength. Although aging is seen as the main factor of sarcopenia, cancer can also be a generator of it. As a poor prognostic factor of cancer, sarcopenia is currently attracting people’s attention. Patients’ GC is usually related to eating disorders, leading to weight and muscle loss; thus, the particular importance of sarcopenia for GC is obvious.

CircRNA plays an important role in tumorigenesis and metastasis, so it can be used as a therapeutic target for cancer. CircITGA7 has an inhibitory effect in colorectal cancer and is a potential target treatment for CRC. The upregulation of circITGA7 in patients with thyroid cancer plays a regulatory role in thyroid cancer and is a potential marker for the diagnosis or progression of thyroid cancer. However, there is no report about the mechanism of circITGA7 in GC. qRT-PCR assay showed that the expression level of circITGA7 was obviously lower in GC tissues and cells than that of the normal ones. Functional studies showed that overexpression of circITGA7 significantly inhibited the progression of AGS and 7,910 cell lines, whereas knockdown of circITGA7 obviously promoted GC cells’ proliferation, invasion, and migration. In summary, circITGA7 showed a strong tumor suppressor activity in GC cells.

Increasing reports show circRNAs can modulate the expression of target mRNA by binding to miRNAs and participate in a variety of life activities (Rong et al., 2017). On the basis of bioinformatics analysis, we screened the miRNA library of circITGA7 and found miR-1471 was directly modulated by circITGA7, whereas the expression level of circITGA7 was suppressed by endogenous miR-1471 level. MiR-1471 is reported to modulate cell proliferation and motility. MiR-1471 overexpression has been implicated in many human cancers (Meng et al., 2018). Bioinformatics analysis and luciferase reporter analysis confirmed miR-1471 was the target of circITGA7. In addition, miR-1471 expression was increased in GC cells after circITGA7 knockdown, confirming our hypothesis. However, the role of miR-1471 in GC has been rarely researched. Our results revealed the importance of the interaction between circITGA7 and miR-1471 in the genesis and development of GC.

In addition, miRNAs play a key role in biological and pathological processes by mediating targets (Tomankova et al., 2012; Zhao et al., 2012). Bioinformatics analysis and luciferase reporter analysis showed miR-1471 was targeted to GC cells by MTDH. MTDH is an oncogene that is overexpressed in many types of malignant tumors. Studies in breast cancer have found that MTDH promotes breast cancer cell proliferation and tumorigenesis by activating multiple signaling pathways. MTDH acts as an oncogene in prostate cancer and is related to the poor prognosis of patients. The expression of MTDH and that of VEGF are related to tumor angiogenesis and progression and are valuable prognostic factors for patients with triple-negative breast cancer. The combination of MTDH inhibition and chemotherapy has proven to have significant efficacy in eliminating human hepatocellular carcinoma xenografts in nude mice, indicating that the development of effective MTDH inhibition strategies will enable objective responses and survival benefits in patients with advanced hepatocellular carcinoma. We hypothesized circITGA7 could suppress GC cells’ proliferation and metastasis by regulating MTDH’s mRNA level through competitive sponge miR-1471. Our result proved overexpression of circITGA7 in tumors could absorb more miR-1471, leading to a reduction of miR-1471 and miRNA-mediated attenuation of MTDH mRNA, thus suppressing invasive tumor growth.

In summary, by using the TCGA data set and comprehensive bioinformatics analysis (GO, KEGG, and PPI network), we identified the key genes related to GC. Our findings suggest circITGA7 is a tumor suppressor regulator. It induces competitive binding to miR-1471, leading to upregulation of MTDH. Our study confirms for the first time that circITGA7 inhibited the occurrence and development of GC by regulating the miR-1471/MTDH axis. Downregulation of circITGA7 is involved in gastric carcinogenesis by regulating miR-1471 axis-mediated MTDH. This study provides a significant basis for further elucidation of the biological characteristics of GC. To verify the role of circITGA7/miR-1471/MTDH axis in the GC, in future studies, we will collect more clinical samples to explore circITGA7/miR-1471/MTDH expression and clinical parameters (including clinical stage, age, and survival time).

## Data Availability Statement

The datasets presented in this study can be found in online repositories. The names of the repository/repositories and accession number(s) can be found in the article/supplementary material.

## Ethics Statement

The studies involving human participants were reviewed and approved by the Ethics Committee of the 980th Hospital of the PLA Joint Logistics Support Force. The patients/participants provided their written informed consent to participate in this study.

## Author Contributions

HJ and BZ contributed to the conception and design. All authors contributed to analysis and interpretation of data, writing, review, and/or revision of the manuscript.

## Conflict of Interest

The authors declare that the research was conducted in the absence of any commercial or financial relationships that could be construed as a potential conflict of interest.

## Publisher’s Note

All claims expressed in this article are solely those of the authors and do not necessarily represent those of their affiliated organizations, or those of the publisher, the editors and the reviewers. Any product that may be evaluated in this article, or claim that may be made by its manufacturer, is not guaranteed or endorsed by the publisher.

## Footnotes

1
https://www.cancer.gov/about-nci/organization/ccg/research/structural-genomics/tcga

2
http://www.mirbase.org/

3
https://circinteractome.nia.nih.gov/

4
http://www.targetscan.org/vert_72/
